# Functional and self-care capacity of people with multiple sclerosis

**DOI:** 10.1590/1518-8345.3068.3183

**Published:** 2019-10-07

**Authors:** Ana Railka de Souza Oliveira-Kumakura, Larissa Maria Bezutti, Juliany Lino Gomes Silva, Renata Cristina Gasparino

**Affiliations:** 1 Universidade Estadual de Campinas, Faculdade de Enfermagem, Campinas, SP, Brazil.

**Keywords:** Activities of Daily Living, SelfCare, Multiple Sclerosis, Human Activities, Chronic Disease, Nursing, Atividades Cotidianas, Autocuidado, Esclerose Múltipla, Atividades Humanas, Doença Crônica, Enfermagem, Actividades Cotidianas, Autocuidado, Esclerosis Múltiple, Actividades Humanas, Enfermedad Crónica, Enfermería

## Abstract

**Objective:**

describe the self-care and functionality levels of patients with multiple sclerosis and determine whether sociodemographic, clinical and functional variables interfere with self-care and/or functionality.

**Method:**

correlational, cross-sectional study with a quantitative approach performed with individuals in outpatient follow-up. We collected sociodemographic and clinical data and applied the Appraisal of Self-care Agency Scale, the Barthel index, the Lawtton and Brody Scale, and the instrument to investigate the performance in Advanced Activities of Daily Living. We performed descriptive and inferential analysis.

**Results:**

most patients were classified as “having self-care” (82.14%); with moderate dependence (51.19%) for the basic activities of daily living, partial dependence for the instrumental activities of daily living (55.95%), and more active for the advanced activities of daily living (85.71%). Patients with longer disease duration had a higher number of disabilities and, in those with better socioeconomic and educational profile, the functionality was better.

**Conclusion:**

disease duration was strongly correlated with a higher number of disabilities and better socioeconomic and educational profiles showed to be protective factors for functionality. Care planning should consider the needs observed by the multidisciplinary team, stimulating the development of self-care, functionality and sociability.

## Abstract (pt)

**Objetivo:**

descrever os níveis de autocuidado e funcionalidade dos pacientes com esclerose múltipla e verificar se as variáveis sociodemográficas, clínicas e funcionais interferem no autocuidado e/ou funcionalidade.

**Método:**

estudo correlacional, transversal, com abordagem quantitativa, realizado com indivíduos em seguimento ambulatorial. Coletamos dados sociodemográficos e clínicos e aplicamos a Escala de Avaliação da Competência para o Autocuidado, o Índice de Barthel, a Escala de Lawton e Brody e o instrumento para investigar a realização de Atividades Avançadas da Vida. Realizamos análise descritiva e inferencial.

**Resultados:**

a maior parte dos pacientes foi classificada como “Tendo autocuidado” (82,14%); com moderada dependência (51,19%) para as atividades básicas da vida diária, dependência parcial para as instrumentais (55,95%) e mais ativos para as avançadas (85,71%). Os pacientes com a doença há mais tempo apresentaram maior número de incapacidades e, naqueles com melhor perfil socioeconômico e educacional, a funcionalidade encontrava-se melhor.

**Conclusão:**

a duração da doença esteve fortemente correlacionada a um maior número de incapacidades e os melhores perfis socioeconômico e educacional mostraram-se fatores protetores para a funcionalidade. O planejamento do cuidado deve levar em consideração as necessidades observadas pela equipe multidisciplinar, estimulando o desenvolvimento do autocuidado, a funcionalidade e sociabilidade.

## Abstract (es)

**Objetivo:**

describir los niveles de autocuidado y funcionalidad de los pacientes con esclerosis múltiple y verificar si las variables sociodemográficas, clínicas y funcionales interfieren en el autocuidado y/o funcionalidad.

**Método:**

estudio correlacional, transversal, con enfoque cuantitativo realizado con individuos en seguimiento de un ambulatorio. Se recogieron datos sociodemográficos y clínicos y fueron aplicadas la Escala de Evaluación de la Competencia para el Autocuidado, el Índice de Barthel, la Escala de Lawtton y Brody y el instrumento para investigar la realización de Actividades Avanzadas de la Vida Diaria. Se realizó un análisis descriptivo e inferencial.

**Resultados:**

la mayor parte de los pacientes fue clasificada como “Realiza autocuidado” (82,14 %); con moderada dependencia (51,19 %) para las actividades básicas de la vida diaria, dependencia parcial para las instrumentales (55,95 %) y más activos para las avanzadas (85,71 %). Los pacientes con mayor duración de la enfermedad presentaron un mayor número de incapacidades y, en aquellos con mejor perfil socioeconómico y educativo, la funcionalidad se encontraba mejor.

**Conclusión:**

la duración de la enfermedad estuvo fuertemente correlacionada con un mayor número de incapacidades y los mejores perfiles socioeconómico y educativo se revelaron factores favorables a la funcionalidad. La planificación del cuidado debe tener en cuenta las necesidades observadas por el equipo multidisciplinario, estimulando el desarrollo del autocuidado, la funcionalidad y la sociabilidad.

## Introduction

Multiple sclerosis (MS) is a chronic inflammatory disease of the central nervous system (CNS), with an autoimmune, demyelinating character that leads to defects in the conduction of synapses. This results in motor and sensory deficits that can last for days or weeks and are completely or partially reversible. Thus, the evolution is characterized by attacks, acute recurrences or follows a progressive clinical course^(^ 1 ^-^ 4 ^)^.

The disease affects about 2.5 million people in the world, in the age group from 20 to 45 years, mostly women, with a higher prevalence in temperate regions^(^ 2 ^-^ 3 ^)^. In Brazil, although the distribution is still not well known, the Southeast region has a higher prevalence, of 15 cases/100,000 habitants^(^ 5 ^-^ 6 ^)^. The etiology is unknown, but it is suggested that it is a multifactorial event, caused by genetic predisposition, autoimmune disease, environmental factors, emotional and/or psychological stress^(^ 1 ^-^ 4 ^)^.

Typically there may be numerous restrictions for the individual, initiated with uncontrollable fatigue or weakness, which may be followed by paresis or hemiparesis, spasticity, gait alteration, incoordination of movements and involuntary tremors. The most common symptoms are monocular visual loss due to optic neuritis, double vision caused by brain stem dysfunction, sensory loss because of transverse myelitis and/or ataxia due to a cerebellar lesion^(^ 1 ^-^ 8 ^)^.

Other manifestations are cognitive dysfunction, in which there are difficulties to maintain attention, alterations in verbal fluency, decreased information processing capacity and memory problems. There may also be alterations in language (dysarthria and/or aphasia), vesical symptoms (alterations in frequency/urgency), intestinal symptoms (constipation more often), as well as autonomic dysreflexia that can lead to cardiovascular alterations, thermal deregulation, or heat sensitivity^(^ 7 ^-^ 8 ^)^. Other conditions that may not be visible to healthcare professionals include anxiety, stress, depression, pain, altered sleep patterns, and sexual dysfunction^(^ 1 ^-^ 8 ^)^.

Considering this context, it is important to discuss the rehabilitation of individuals with MS, because it is an incapacitating disease that gradually affects the person’s self-care capacity and causes functional decline, evidenced by the evaluation of basic, instrumental, and advanced activities of daily living. Such activities, if compromised, may have an impact not only on self-care, but also on quality of life, social interaction, and may contribute to the isolation of the individual^(^ 9 ^-^ 10 ^)^.

Self-care is defined by the World Health Organization (WHO) as “the capacity of individuals, families and communities to promote health, prevent disease, maintain health, and deal with diseases and disabilities with or without support from a health care provider”^(^ 11 ^)^. Functionality, according to the International Classification of Functioning, Disability and Health (ICF), reflects the interaction between the health condition and the environmental and personal context of the individual and depends on the preservation of autonomy and independence, which are ensured by the preservation of cognition, humor, mobility, and communication^(^ 12 ^)^. This evidences that the relation between these constructs is bidirectional: functional capacity affects self-care and vice-versa.

In this context, nurses have a fundamental role in integrating the multidisciplinary team and ensuring the promotion, protection, and rehabilitation of health, focusing on the maintenance of self-care and on the functionality of these patients. However, we noticed a shortage of scientific publications, including nursing, to address this issue. Therefore, the objectives of the study were to describe the levels of self-care and functionality of patients with multiple sclerosis and to verify if sociodemographic, clinical, and functional variables interfere with self-care and/or functionality. We believe that this study may contribute to improve the care, the professionals’ understanding about functionality and self-care, and to the development of rehabilitation services.

## Method

Correlational, quantitative, and cross-sectional study carried out in outpatient clinics of a public tertiary hospital in the city of Campinas, São Paulo, Brazil, from October to December 2017. We included patients regardless of disease diagnosis time, aged above 18 years, and excluded those with other neurological diseases. The sample size was calculated with the objective of evaluating the correlation between functionality and self-care by Pearson’s correlation coefficient. We assumed a test power of 80%, a significance level of 5%, an estimate for the correlation coefficient equal to 0.30, which can be considered a coefficient of average degree, and a correlation coefficient equal to 0.00 as null hypothesis^(^ 13 ^)^. The calculation resulted in a minimum sample of 84 patients. To perform the sample calculations we used the G*Power 3.1.9.2. software.

An undergraduate nursing student, in the last year of the course, collected the data. She received 8-hour training, which included theoretical and practical aspects for the application of the scales, from the main researcher, advisor for the study. The other assistant researchers contributed with the analysis and interpretation of the results.

The collection instrument included sociodemographic data (age, sex, family income, city of birth, professional and civil situation) and clinical data (type of MS, medications in use, clinical manifestations) collected directly from patients through interviews. We applied the following scales translated and validated for use in Brazil: Appraisal of Self-care Agency Scale - Revised (ASA-R)^(^ 14 ^-^ 15 ^)^, Lawton and Brody Scale (LBS)^(^ 16 ^)^, and Barthel Index (BI)^(^ 17 ^)^. We also used the instrument to investigate the performance of Advanced Activities of Daily Living (AADLs)^(^ 18 ^)^.

To supplement the evaluation, we collected from the medical chart the Kurtzke Expanded Disability Status Scale (EDSS) score attributed by the neurologist during the consultation of the day. The EDSS is a medical scale that evaluates the level of disabilities clinically observed of the individual with MS. It is divided into eight functional systems (pyramidal, cerebellar, brain stem, sensory, intestine and bladder, visual, cerebral and others) and based on these indicators a score is attributed that ranges from 0 (normal) up to 10 (death by MS). Higher scores reflect a higher level of disability^(^ 19 ^)^.

The ASA-R is based on Dorothea Orem’s Self-Care Deficit Theory and evaluates a human being’s ability to perform the practices in the care of oneself, in the relation between the individual and the environment^(^ 20 ^)^. It is a Likert-type scale with 15 items and five answer options (I totally disagree, I disagree, I do not know, I agree, and I totally agree) and presents three possible results named as: “Having capacity for self-care, Developing capacity for self-care, and Lacking capacity for self-care.” The possible interval for the total measure ranges from 15 to 75 points, and higher values reflect higher self-care capacity^(^ 14 ^-^ 15 ^,^ 20 ^)^.

The LBS evaluates the functional condition for instrumental activities of daily living (IADL) graded as to the degree of assistance required for each activity, namely: use telephone, shop, prepare meal, perform domestic activities, use means of transport, take medications, and manage finance^(^ 14 ^)^. The score for each item ranges from 1 to 3 and, for global interpretation, the scale was converted into three groups: equal to or lower than 7 means total dependence; from 7 to 20 corresponds to partial dependence; and equal to 21 expresses independence^(^ 21 ^)^.

The BI evaluates the basic activities of daily living (BADLs) and aims to evaluate independence (physical or verbal) in personal care, mobility, locomotion, and eliminations. It has ten items and each one is scored according to the patient’s performance. The score ranges from 0 to 100: a total of 0-20 indicates total dependence; 21-60, severe dependence; 61-90, moderate dependence; 91-99, slight dependence; and 100, independence^(^ 17 ^,^ 22 ^)^.

To evaluate the AADLs we used the same instrument used in the longitudinal study Health, Welfare and Aging (SABE). The AADLs comprise 12 social, productive, physical and leisure activities that involve superior cognitive functions, which are: (1) contact with other people by means of letters, telephone or email; (2) visiting friends and family members at their homes; (3) care or assistance to other people (including personal care, transportation, purchases for family or friends); (4) voluntary work outside the home; (5) trip out of the city spending at least one night out; (6) participation in any regular exercise program (e.g. sports, physical exercises, walks and corporal practice groups); (7) inviting people to come to your home for meals or leisure; (8) go out with other people to public places such as restaurant or cinema; (9) conducting some manual activity, crafts or artistic activity; (10) participation in organized social activities (clubs, community or religious groups, elderly coexistence centers, bingo); (11) using computer, including the Internet; (12) driving motor vehicles^(^ 18 ^)^.

The questions for evaluating the AADLs were answered through a scale with five answer options (always, often, occasionally, rarely, and never). The answers: always, often, and occasionally were considered as performance of the activity and received value 1. The total score ranged from 0 to 12, being classified as “more active” those who performed five or more activities^(^ 18 ^)^.

We used descriptive statistics with frequency, dispersion measures and central tendency. We applied the Shapiro-Wilk test to check the normality of the numerical data. For comparisons involving a qualitative variable with two categories and one quantitative variable, we applied the non-parametric Mann-Whitney test or the unpaired student’s t-test, according to the data distribution. For comparisons involving one qualitative variable with more than two categories and one quantitative variable, we applied the non-parametric Kruskal-Wallis test, followed by Dunn’s post-test^(^ 23 ^-^ 24 ^)^.

We applied the Spearman correlation coefficient to check the correlations between the quantitative variables and used the following classification: 0.1 to 0.29 (weak correlation), 0.30 to 0.49 (moderate correlation), and greater than or equal to 0.50 (strong correlation)^(^ 13 ^)^. To study the associations between the qualitative variables, we applied the Pearson’s chi-squared test and, for the cases in which the assumptions were not met, we used Fisher’s exact test^(^ 23 ^-^ 24 ^)^. For all analyses we used the statistical software SAS version 9.4 and SPSS version 24.0.

The project was approved by the Research Ethics Committee of the University of Campinas, no. 2,305.539 and all recommendations of Resolution 466/2012 referring to research with human beings were complied with. The manuscript followed the recommendations of the guide Revised Standards for Quality Improvement Reporting Excellence (SQUIRE 2.0)^(^ 25 ^)^.

## Results

Patients with MS under outpatient follow-up were predominantly female (71.4%), with mean age of 40.2 years (SD=11.7), born (88.1%) and living (96.4%) in the state of São Paulo, married (74.8%), with complete secondary education (28.6%) or complete higher education (27.4%). They had mean family income of 3.9 minimum wages and lived with two persons at home. Regarding the professional status, 70.2% were inactive.

Clinically, relapsing-remitting MS (RRMS) predominated (84.5%). The symptoms onset was on average 11.8 years prior (SD=8.1), the diagnosis time was 9.1 years (SD=6.8), and the mean EDSS score was 3.9 points (SD=2.3). Regarding the clinical manifestations, we highlight the pyramidal (89.3%), disorders of mental/emotional functions (79.8%), cerebellar alterations (76.2%), report of fatigue (69.0%), vesical disorders (48.8%) and intestinal disorders (48.8%). Regarding the medications in use, we found mainly Interferon (33.3%) and Natalizumab (26.2%).

Table 1 presents the results of the self-care and functioning evaluation scores.

**Table 1:** Characterization of the self-care and functioning evaluation scores of patients with multiple sclerosis (n=84). Campinas, SP, Brazil, 2017

Variables	n (%)	Mean (SD)*	Median	Variation
Appraisal of Self-care Agency (ASA-R)^†^		53,8 (3,9)	53,5	45,0 - 65,0
Having capacity for self-care	69 (82,1)			
Developing capacity self-care	15 (17,9)			
Classification for BI^‡^		84,0 (18,9)	87,5	5,0 - 100,0
Independent	28 (33,3)			
Slight dependence	8 (9,5)			
Moderate dependence	43 (51,2)			
Severe dependence	3 (3,6)			
Total dependence	2 (2,4)			
Classification for LBS^§^		18,4 (3,6)	20,0	8,0 - 21,0
Independent	37 (44,0)			
Partial dependence	47 (55,9)			
Classification for AADL^||^		6,6 (2,9)	6,5	1,0 - 12,0
More active	72 (85,7)			
Less active	12 (14,3)			

* SD = Standard Deviation;

† ASA-R = Appraisal of Self-care Agency Scale - Revised;

‡ BI = Barthel index;

§ LBS = Lawton and Brody Scale;

|| Advanced Activities of Daily Living

We found that most patients were classified as “having self-care” (82.1%); with moderate dependence (51.2%) for BADLs; partial dependence for IADLs (55.9%); and more active for AADLs (85.7%) (Table 1).

When evaluating the influence of functioning and sociodemographic measures on the ASA-R, we found no correlation between the variables (Table 2). By investigating in isolation the influence of clinical variables on patient self-care, we found that only the presence of pyramidal manifestations interfered with this measure (p=0.0281).

**Table 2:** Comparison of the sociodemographic and clinical variables with the self-care and functioning scores. Campinas, SP, Brazil, 2017

Variables	ASA-R*	BI^†^	LBS‡	AADL^§^
Age				
ρ^||^	-0,0787	-0,4937	-0,3929	-0,4054
p-value^¶^	0,4770	<0,0001	0,0002	0,0001
N	84	84	84	84
Income				
ρ^||^	-0,1738	0,0489	0,2226	0,2371
p-value^¶^	0,1208	0,6646	0,0458	0,0331
N	81	81	81	81
Diagnosis time				
ρ^||^	-0,0724	-0,3282	-0,2037	-0,2609
p-value^¶^	0,5127	0,0023	0,0631	0,0165
N	84	84	84	84
Symptoms onset time				
ρ^||^	-0,1210	-0,4794	-0,2631	-0,1879
p-value^¶^	0,2729	<0,0001	0,0156	0,0869
N	84	84	84	84
Outpatient follow-up time				
ρ^||^	-0,0238	-0,3692	-0,2066	-0,1583
p-value^¶^	0,8319	0,0006	0,0626	0,1554
n	82	82	82	82
EDSS** score				
ρ^||^	-0,0388	-0,7832	-0,5728	-0,5166
p-value^¶^	0,7262	<0,0001	<0,0001	<0,0001
n	84	84	84	84

* ASA-R = Appraisal of Self-care Agency Scale - Revised;

† BI = Barthel index;

‡ LBS = Lawton and Brody Scale;

§ AADL = Advanced Activities of Daily Living;

|| ρ = Spearman Correlation Coefficient;

¶ p-value = level of significance;

** EDSS = Kurtzke Expanded Disability Status Scale

Through analysis of the correlations between numerical variables and functioning scales scores, we found that age and EDSS score showed negative correlation with all measures. In addition, the symptoms onset time, diagnosis time, and outpatient follow-up time variables also negatively influenced at least one of these measures (Table 2). The self-care scale score was not influenced by any numerical variable.

By examining the comparison measures, through analysis of the functioning and self-care measures categorized, we found that the younger people who had shorter diagnosis time or outpatient follow-up time were more functional. Patients with lower EDSS scores were more independent for BADLs, IADLs, and AADLs, and those with higher income were more active for AADLs (Table 3).

**Table 3:** Comparison of the sociodemographic and clinical variables with the groups identified by the evaluation of functioning (n=84). Campinas, SP, Brazil, 2017

Variables		n	Mean (SD*)	Median	Variation	p-value^†^
	**BI** ^‡^					
Age	Independent/Slight	36	33,2 (9,8)	31,0	18,0 - 61,0	< 0,001
	Moderate/Severe/Total	48	45,5 (10,2)	46,5	26,0 - 66,0	
Diagnosis	Independent/Slight	36	6,7 (5,5)	5,0	0,0 - 20,0	0,010
	Moderate/ Severe/Total	48	10,8 (7,2)	9,5	0,3 - 26,0	
Symptoms onset	Independent/Slight	36	7,9 (6,0)	7,0	0,3 - 20,0	0,001
	Moderate/Severe/Total	48	14,8 (8,3)	16,0	1,0 - 33,0	
Outpatient follow-up	Independent/Slight	35	5,8 (5,3)	4,0	0,0 - 17,0	0,005
	Moderate/Severe/Total	47	10,4 (7,4)	9,0	0,0 - 26,0	
EDSS^§^ score	Independent/Slight	36	2,1 (1,4)	2,0	0,0 - 6,0	< 0,001
	Moderate/Severe/Total	48	5,4 (1,7)	6,0	2,0 - 8,0	
	**LBS** ^||^					
Age	Independent	37	36,2 (9,2)	34,0	23,0 - 57,0	0,004
	Partially dependent	47	43,4 (12,6)	45,0	18,0 - 66,0	
EDSS score	Independent	37	2,8 (1,9)	2,0	0,0 - 6,0	< 0,001
	Partially dependent	47	4,9 (2,1)	5,5	1,0 - 8,0	
	**AADL** ^††^					
Age	More active	72	38,8 (10,9)	37,0	18,0 - 62,0	0,014
	Less active	12	48,5 (13,0)	48,5	23,0 - 66,0	
Income	More active	69	3702,9 (2500,5)	3000,0	700,0 - 12000,0	0,011
	Less active	12	3658,3 (6770,3)	1550,0	600,0 - 25000,0	
Diagnosis	More active	72	8,4 (6,6)	7,0	0,0 - 26,0	0,018
	Less active	12	13,2 (6,6)	11,5	1,0 - 23,0	
EDSS^§^ score	More active	72	3,7 (2,2)	3,2	0,0 - 8,0	0,020
	Less active	12	5,4 (2,1)	6,0	1,5 - 7,5	

* SD = Standard Deviation;

† p-value = level of significance;

‡ BI = Barthel Index;

§ EDSS = Kurtzke Expanded Disability Status Scale;

|| LBS = Lawton and Brody Scale;

^¶^AADL = Advanced Activities of Daily Living

Table 4 shows that the higher the educational level, the greater the independence in relation to all activities of daily living. By applying Dunn’s post-test, we observed that this difference was significant when comparing patients with elementary education and patients with complete higher education as to the three measures of functioning. In addition, patients with preserved functioning maintained an active professional status. While those with clinical manifestations of the disease presented greater dependence for all activities.

**Table 4:** Comparison between the functioning scores and the sociodemographic and clinical variables of patients with multiple sclerosis (n=84). Campinas, SP, Brazil, 2017

Variables	n	Mean (SD*)	Median	Variation	p-value^†^
**BI** ^‡^					
Educational level					
Elementary education	21	75,5 (20,8)	80,0	25,0 - 100,0	0,016
Secondary education	29	85,7 (14,7)	90,0	45,0 - 100,0	
Higher education	34	87,9 (19,8)	95,0	5,0 - 100,0	
Professional status					
Active	25	90,8 (16,4)	100,0	45,0 - 100,0	0,003
Inactive	59	81,2 (19,4)	85,00	5,0 - 100,0	
Cerebellar manifestation					
Present	64	81,1 (19,5)	85,0	5,0 - 100,0	0,001
Absent	20	93,5 (13,5)	100,0	50,0 - 100,0	
Vesical/intestinal manifestation					
Present	41	75,8 (21,2)	80,0	5,0 -100,0	< 0,001
Absent	43	91,9 (12,4)	100,0	50,0 - 100,0	
**LBS** ^§^					
Educational level					
Elementary education	21	16,4 (4,9)	19,0	8,0 - 21,0	0,045
Secondary education	29	18,9 (2,6)	20,0	12,0 - 21,0	
Higher education	34	19,3 (3,1)	21,0	8,0 - 21,0	
Professional status					
Active	25	19,9 (2,3)	21,0	11,0 - 21,0	0,007
Inactive	59	17,8 (3,9)	19,0	8,0 - 21,0	
Cerebellar manifestation					
Present	64	17,8 (3,8)	19,0	8,0 - 21,0	0,001
Absent	20	20,2 (2,3)	21,0	11,0 - 21,0	
Vesical/intestinal manifestation					
Present	41	17,5 (3,9)	19,0	8,0 - 21,0	0,003
Absent	43	19,3 (3,1)	21,0	8,0 - 21,0	
**AADL** ^||^					
Educational level					
Elementary education	21	4,9 (2,9)	5,0	1,0 - 10,0	0,008
Secondary education	29	6,9 (2,4)	7,0	2,0 - 10,0	
Higher education	34	7,5 (2,9)	8,0	2,0 - 12,0	
Professional status					
Active	25	8,2 (2,5)	9,0	3,0 - 12,0	0,001
Inactive	59	5,9 (2,8)	6,0	1,0 - 12,0	
Cerebellar manifestation					
Present	64	6,2 (2,9)	6,0	1,0 - 12,0	0,020
Absent	20	7,9 (2,5)	8,5	2,0 - 11,0	

* SD = standard deviation;

† p-value = level of significance;

‡ BI = Barthel Index;

§ LBS = Lawton and Brody Scale;

|| AADL = Advanced Activities of Daily Living

## Discussion

Our results showed the implications of MS on self-care and on the basic, instrumental, and advanced activities of daily living of patients. Such evidence corroborates previous studies that sought to explore the changes in the disabilities according to the severity of the disease^(^ 26 ^-^ 28 ^)^. The manifestations of MS are highly variable ​and often unpredictable, with its course evolving from mild and infrequent relapses, with limited impact on functional capacity, to rapidly accumulating disability, loss of independent ambulation, and extensive cognitive impairment^(^ 29 ^)^.

Regarding the profile of our patients, there was predominance of females and with mean age of 40.2 years and with high educational level, in line with national studies^(^ 30 ^-^ 31 ^)^ and international studies^(^ 27 ^)^. We highlight that the age of our group ranged from 18 years to 66 years. We found in the literature that most patients are diagnosed at the beginning of adulthood, between 20 and 40 years of age, that the disease is twice as prevalent among women, and that higher educational level is protective against the cognitive function decline and disease overload^(^ 32 ^-^ 33 ^)^. This demonstrates that MS causes neurological disability in young adults^(^ 30 ^)^, leading to serious adverse effects on the daily living of many patients, including the loss of capacity for work^(^ 29 ^)^.

However, it is worth adding that some studies have pointed out that women, individuals with higher education, and youth with MS have greater self-management of the disease^(^ 34 ^-^ 35 ^)^. This term is defined as an active process of dealing with the disease through adherence to treatment, participation in therapeutic decision-making, active search for information about the disease and new treatment options, maintenance of social relationships and emotional balance, that is, it incorporates self-monitoring and symptom management. Therefore, self-management is part of self-care in order to facilitate better adaptation to the disease, reduce the likelihood of secondary complications, contribute to quality of life, reduce disability, and improve outcomes with treatment^(^ 34 ^,^ 36 ^)^.

As the treatment schemes for MS can be complex, patients with low levels of literacy may have greater difficulty to understand and complying with the treatment. This may pose a challenge to care and lead to self-care impairment and cause functional disability, as we observed.

Contributing to the characterization of this public, we found that most of the patients were inactive and that the family income was 3.9 minimum salaries, today approximately BRL 3,720^(^ 29 ^)^. In addition, we found that patients with higher income were more active for AADLs and those with preserved functioning maintained an active professional status.

A study conducted in Sweden, which compared salaries between the general population and patients with MS before and after the diagnosis of the disease, found that the gross salary was similar before, but shortly after the diagnosis of the disease it diminished. After eleven years of follow-up, people without disease had an increase of 7,520 euros in their salaries, while patients with MS had a loss of 9,010 euros, which represents more than 30% of the mean annual gross salary in Sweden^(^ 29 ^)^. Moreover, since the initial symptoms of MS appear during the economically active age of a person, this may impact the ability to maintain a job; however, rates for unemployment and retirement due to disability vary between countries^(^ 37 ^-^ 39 ^)^.

The literature shows that the treatment of MS is costly, which makes many people unable to afford it, delaying or preventing the search for medical treatment^(^ 27 ^)^. Often, patients cannot afford the medical care expenses and because of that do not attend the consultations or do not undergo the diagnostic exams or, even, omits their symptoms in an effort to reduce costs^(^ 27 ^)^. This may have caused the inconsistency between the symptoms onset time (11.8 years) and the diagnosis time (9.1 years). It is worth noting that the time between the clinical onset of MS and the diagnosis is the most important predictor of the severity of disability^(^ 40 ^)^.

The clinical manifestations that predominated in this study were the pyramidal (paresis and paresthesia), cerebellar (ataxia and dysarthria) and vesical-intestinal ones. They are often originated after an outbreak of the disease and progress from a transient symptom to a sequela. However, many patients feel discouraged as to seeking traditional care, in which there is a delay in scheduling and, consequently, in feedback^(^ 27 ^)^.

In addition, there is the fact that we work with individuals of the Y and Z generations, born in the Digital age, in which communication is practically instantaneous. In this context, they increasingly seek for clinics with more affordable prices, emergency room, or search for information on the Internet. Such practices may result in a delay in the diagnosis and correct treatment. It should be emphasized the importance of effective care, due to the complexity of the disease, as well as its chronic nature^(^ 27 ^)^.

It is found in the literature that the aging process leads to cerebral trophic decrease, which contributes to the neurodegeneration process and decreases remyelination. The compact myelination of the cerebral white matter ends at 40 years of age and begins to degenerate in the following years. Therefore, the more limited recovery after the clinical outbreaks of the disease, in the first five years, can cause progression of MS and lead to sequelae, loss of self-care, and decreased functioning^(^ 41 ^)^. Moreover, the decline in functional reserve produces the insidious accumulation of physical and cognitive disability, which characterizes what is traditionally considered as the progressive phase of the disease^(^ 4 ^)^.

Thus, patients with greater clinical compromise and with longer symptoms onset time and/or diagnosis time showed higher levels of dependence. This result is evidenced in another study conducted in the city of Ankara, Turkey, in which the researchers confirmed that, in the first 10 years of the disease, patients tend to be slightly more dependent to perform the activities of daily living and with a greater need for self-care^(^ 27 ^)^.

It should be noted that, in the present study, most patients with MS were classified as “having capacity for self-care” and presented higher dependence to perform the basic activities, mainly for sphincter control, when compared with the instrumental or advanced activities of daily living. As for self-care, only the presence of pyramidal sequelae interfered with this measure.

The clinical course of multiple sclerosis is difficult to be predicted in the different groups and individuals. A study that discusses the topographic models of MS, as a new approach to characterize the clinical course of the disease, reports that the location of the lesion defines the clinical symptoms and the functional systems affected: those on the spinal cord produce pyramidal, sensory, intestinal, and bladder symptoms; those on the brain stem and cerebellum cause diplopia, vertigo, ataxia and imbalance; and those on the hemispheres produce brain signals, most notably cognitive dysfunction. In light of that, they reported that spinal cord lesions, in particular, have shown worse prognosis and loss of functional reserve, which will limit their self-care in advance^(^ 4 ^)^.

This reinforces the idea that biopsychosocial mechanisms are associated with the level of disability. Since MS is a neurodegenerative disease, it affects neural structures, which hinders or even prevents the transmission of stimuli to the other systems and organs of the body, and this has direct impact on cognition, sphincter control, manual skills, mobility, transfer, and is evident in difficulties to perform the basic and instrumental activities of daily living^(^ 26 ^,^ 42 ^)^. These factors should be considered in the survey of nursing diagnoses performed by nurses^(^ 30 ^,^ 43 ^)^.

A study aimed at implementing the Systematization of Nursing Care for outpatient follow-up of patients with MS found as the most frequent nursing diagnoses, according to NANDA International, Inc. (NANDA-I), impaired physical mobility (00085), sleep pattern disorders (00198), self-care deficits, activity intolerance (00092), impaired urinary elimination (00016), risk for ineffective personal coping (00069), constipation (00011), impaired memory (00131), sexual dysfunction (00059), ineffective control of therapeutic regimen (00080), and acute pain (00132)^(^ 10 ^)^.

Often times, after the onset of demyelinating diseases, psychiatric disorders may arise^(^ 8 ^)^. From this perspective, the maintenance of AADLs, related to social interaction, are beneficial to these patients, because they generate benefits for both physical and psychological health. In addition to improving self-esteem, decreasing social isolation and the onset of depressive disorders^(^ 30 ^)^.

Some studies have also pointed out the protective effect of AADLs by decreasing cognitive declines and the onset of dementia, avoiding the decline of memory and language, executive functions, attention, and global cognitive functions^(^ 8 ^,^ 44 ^)^. Therefore, nurses should act since the onset of the first clinical manifestations, by providing emotional support to the person, who will possibly be shaken, due to the possible disabilities and the changes that will occur not only in the body, but also in the lifestyle^(^ 8 ^,^ 26 ^)^. This may be conducted through motivation to preserve self-care activities, as well as through support and suggestion of alternatives for the functional disabilities^(^ 10 ^,^ 30 ^)^.

The planning of care, as well as its implementation must be consistent with the needs observed, considering, mainly, the time of manifestation of the disease, since we know that it has a crucial role for functional decline. A beneficial factor is the inclusion of family members in this care plan, which will positively contribute to the promotion and rehabilitation of this patient. This factor may contribute directly by fostering greater willingness to perform all activities of daily living, by stimulating the sociability and the functioning of the individuals^(^ 45 ^)^.

In this context, health professionals should always be attentive to care management, since the deprivation of these activities has a negative effect for these individuals, constituting a possible predictor of social isolation and loss of functioning. Thus, the interaction with the family and society are of fundamental importance to contribute to the promotion and rehabilitation of the patient’s health.

Moreover, the evaluation of the multidisciplinary team is mandatory, since there are countless clinical, functional and self-care demands. In this context, it is important that nurses get involved in a broader way in the promotion and rehabilitation of these patients’ health, working not only in the implementation and systematization of care, but also by stimulating the development of self-care, functioning and sociability. So that the perspective in relation to the individual reaches far beyond the outpatient or bedside scope, with the development of new researches and more and better professional training for these demands that accompany patients with chronic-degenerative diseases.

We highlight some limitations of the present study as to not finding people classified as having greater need for self-care, in addition to the study design, which, for being cross-sectional, does not enable the establishment of cause and effect relation. Other limitations associated with the use of secondary data, such as the possibility of reporting errors, should also be considered. Furthermore, we emphasize the low number of studies on self-care assessment or that study the functioning of people with MS, which made it difficult to compare the results with other findings.

## Conclusion

The results of this study evidenced that the self-care levels of the patients with MS were preserved and 82.1% were classified as “having capacity for self-care.” They presented more pronounced functional limitation in BADLs, if compared with IADLs, 55.9% had partial dependence for IADLs and 51.2%, had moderate dependence for BADLs. As for AADLs, 85.7% were more active. In addition, disease duration was strongly correlated with greater number of disabilities and the best socioeconomic and educational profiles proved to be protective factors for functionality.

This was one of the first studies in Nursing that verified the relation between self-care and functionality of patients with MS, using validated scales and a multidimensional evaluation. We hope that these findings stimulate new researches in the area of rehabilitation in nursing, aiming at the implementation of interventions geared toward the actual needs of the individuals.
